# Supplementary material for: Endosonographic finding of the simultaneous depiction of bile and pancreatic ducts can predict difficult biliary cannulation on endoscopic retrograde cholangiopancreatography
Source: PLoS One. 2020 Jul 9;15(7):e0235757. doi: 10.1371/journal.pone.0235757 (PMC7347092; doi:10.1371/journal.pone.0235757)
Supplement: S1 Table — (DOCX) [file pone.0235757.s001.docx]

**S1 Table. Detailed diagnosis of excluded 204 cases**

|  | Patients who underwent ERCP only | Patients who underwent CLAEUS after ERCP |
| --- | --- | --- |
| N | 204 | 25 |
| Diagnosis  (emergency procedure) |  |  |
| Acute pancreatitis | 6 (4) | 0 (0) |
| Chronic pancreatitis with multiple pancreatic duct stones | 3(2) | 1 (0) |
| Other pancreatic disorders (^a^IPMN, pancreatic trauma) | 0 (0) | 3 (0) |
| Biliary stone and/or sludge | 42 (10) | 4 (0) |
| Bile duct dilatation | 3 (2) | 1 (0) |
| Acute cholangitis | 89 (68) | 9 (6) |
| Acute cholecystitis | 4 (3) | 0 (0) |
| Obstructive jaundice | 13 (5) | 3 (3) |
| Malignant biliary stricture at hilum | 2 (1) | 1 (0) |
| s/p surgical reconstruction | 7 (5) | 2 (0) |
| Other biliary disorders (Lemmel syndrome:1, pancreatobiliary trauma:1, bile leak s/p surgery:3) | 5 (3) | 1 (0)  (Mirrizzi syndrome:1) |
| Non pancreatobiliary disorders (hepatocellular carcinoma, metastatic cancer) | 2 (0) | 0 (0) |
| Abnormal LFT (suspected stones or sludge) | 3 (2) | 0 (0) |

^a^CLAEUS, curved linear array endoscopic ultrasound

^b^ERCP, endoscopic retrograde cholangiopancreatography

^c^IQR, interquartile range

^d^LFT, liver function test
