# Supplementary material for: Endosonographic finding of the simultaneous depiction of bile and pancreatic ducts can predict difficult biliary cannulation on endoscopic retrograde cholangiopancreatography
Source: PLoS One. 2020 Jul 9;15(7):e0235757. doi: 10.1371/journal.pone.0235757 (PMC7347092; doi:10.1371/journal.pone.0235757)
Supplement: S2 Table — (DOCX) [file pone.0235757.s002.docx]

S2 Table. Characteristics of patients undergoing ^a^CLAEUS-naïve ^b^ERCP

|  | All CLAEUS-ERCP patients | Number of excluded cases | Detailed reason for exclusion |
| --- | --- | --- | --- |
| N (%) | 152 | 72 |  |
| Age (median [^c^IQR]) | 70 [59,78] | 68[59,78] |  |
| Male (%) | 80 (52) | 39 (54) |  |
| Emergency procedure |  |  |  |
| Yes (%) | 30 (20) | 19 (26) |  |
| Diagnosis (%) |  |  |  |
| Pancreatic ductal adenocarcinoma | 8 (5) | 4 | ^d^Ph mass adjacent to the ampulla or ampulla lesion |
| Acute pancreatitis | 8 (5) | 3 | Pseudocyst at Ph |
| Other pancreatic disorders | 11 (7) | 9 | Chronic pancreatitis w stones:4  ^e^IPMN:2 ^f^MCN:1  s/p panc surgery and modified anatomy:2 |
| Biliary tract adenocarcinoma | 12 (8) | 6 | Ampulla lesion or ^g^BD lesion adjacent to the ampulla |
| Gallbladder cancer | 2 (1) |  |  |
| Biliary stone and/or sludge | 48 (32) | 17 | Impacted stones at ampulla |
| Acute cholangitis without stone, sludge, mass, or obstruction | 25 (16) | 9 | Severe edema with inflammation at ampulla |
| Obstructive jaundice | 22 (14) | 15 | Anomalous arrangement of ^h^PD/BD:1  No ampulla images available:14  *14 cases were diagnosed with CT scan or ^i^TAUS image of possible abnormal caliber change at biliary tract with abnormal ^j^LFT. |
| Biliary stricture | 6 (4) | 5 | Ampulla stricture with fibrosis:2 |
| Other biliary disorders |  | 2 | Lemmel syndrome,  no ampulla images available:1  Acute cholecystitis,  no ampulla images available:1 |
| Abnormal LFT | 4 (3) | 2 | No apparent abnormality on CLAEUS,  no ampulla images available:2 |

^a^CLAEUS, curved linear array endoscopic ultrasound

^b^ERCP, endoscopic retrograde cholangiopancreatography

^c^IQR, interquartile range

^d^Ph, pancreatic head

^e^IPMN, intrapapillary mucinous neoplasm

^f^MCN, mucinous cystic neoplasm

^g^BD, bile duct

^h^LFT, liver function test

^i^TAUS, transabdominal ultrasound
