# Supplementary material for: Endosonographic finding of the simultaneous depiction of bile and pancreatic ducts can predict difficult biliary cannulation on endoscopic retrograde cholangiopancreatography
Source: PLoS One. 2020 Jul 9;15(7):e0235757. doi: 10.1371/journal.pone.0235757 (PMC7347092; doi:10.1371/journal.pone.0235757)
Supplement: S3 Table — (DOCX) [file pone.0235757.s003.docx]

**S3 Table:**  **Unadjusted and adjusted odds ratios for difficult ^a^SBDC in patients positive for “simultaneous depiction of bile and pancreatic ducts”**

|  | OR (95% CI) | *P* value |
| --- | --- | --- |
| Unadjusted | 14.7 (3.9-54.1) | <0.001 |
| Threshold 1 | 0.31 (0.09-1.04) | 0.057 |
| Threshold 2 | 0.094 (0.028-0.31) | <0.001 |
| Threshold 3 | 0.044 (0.011-0.16) | <0.001 |
| Threshold 4 | 0.056 (0.012-0.25) | <0.001 |

The primary analysis (unadjusted) was performed with the logistic regression model, setting simultaneous depiction, age and sex as an independent variable and difficulty in SBDC defined as cases required more than 20 minutes before successful deep biliary cannulation as a dependent variable.

Adjusted analysis1: adjusted for successful deep biliary cannulation time as more than 5 minutes.

Adjusted analysis2: adjusted for successful deep biliary cannulation time as more than 10 minutes.

Adjusted analysis3: adjusted for successful deep biliary cannulation time as more than 15 minutes.
