# Supplementary figures and images for: Endosonographic finding of the simultaneous depiction of bile and pancreatic ducts can predict difficult biliary cannulation on endoscopic retrograde cholangiopancreatography
Source: PLoS One. 2020 Jul 9;15(7):e0235757. doi: 10.1371/journal.pone.0235757 (PMC7347092; doi:10.1371/journal.pone.0235757)

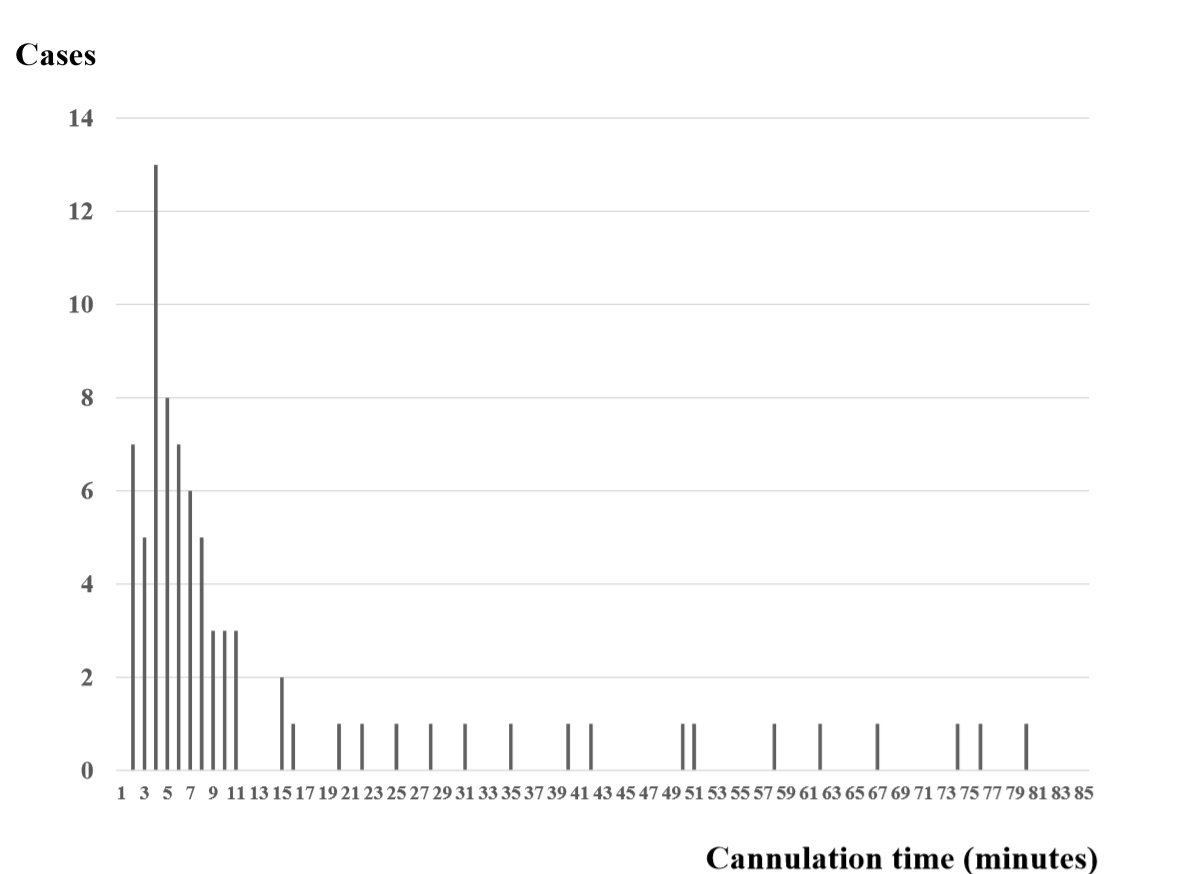

Supplement: S1 Fig — (TIF) [file pone.0235757.s004.tif]
