# Supplementary material for: Endosonographic finding of the simultaneous depiction of bile and pancreatic ducts can predict difficult biliary cannulation on endoscopic retrograde cholangiopancreatography
Source: PLoS One. 2020 Jul 9;15(7):e0235757. doi: 10.1371/journal.pone.0235757 (PMC7347092; doi:10.1371/journal.pone.0235757)
Supplement: Attachment — Submitted filename: Plos1.docx [file pone.0235757.s005.docx]

Shinichiro Ueda, et al: Endosonographic finding of the simultaneous depiction of bile and pancreatic ducts can predict difficult biliary cannulation on endoscopic retrograde cholangiopancratography

The discussed study is a single center, retrospective cohort study, including 80 patients. All patients have curved linear array endoscopic ultrasound (CLAEUS) before endoscopic retrograde cholangiopancreatography (ERCP).

The general aim of the study, was to define predictors of difficult selective bile duct cannulation (SBDC).

Hereby difficult is described as the simultaneous depiction of bile and pancreatic ducts at the second portion of the duodenum farther than 10 mm away from the papilla.

It therefore has been concluded, that if a CLAEUS is done in advance, the risk of a post-ERCP pancreatitis, as well as the risk of complications is reduced.

Limitations of the study:

I find it irritating, that the division of the patients into two groups were on the basis of the duration of the examination, and more importantly, that the used threshold, was define by the study group. Is this threshold robust? It seems to be necessary, that this threshold is varied. Furthermore additional factors in the definition of the threshold should be considered, such as the mentioned number of papilla contacts.

In Addition the number of patients were initially 356, but included in the study were only 80. This strong selected patient group I find very odd and would like a more detailed explanation of why 356 patients were included in the first place.

Decision: major revision
